# Diminished Time-Based, but Undiminished Event-Based, Prospective Memory Among Intellectually High-Functioning Adults With Autism Spectrum Disorder: Relation to Working Memory Ability

**DOI:** 10.1037/neu0000008

**Published:** 2013-10-14

**Authors:** David M. Williams, Christopher Jarrold, Catherine Grainger, Sophie E. Lind

**Affiliations:** 1 Department of Psychology, Durham University, Durham, U.K.; 2 School of Experimental Psychology, University of Bristol, Bristol, U.K.

**Keywords:** autism, prospective memory, working memory, short-term memory (STM), complex span

## Abstract

***Objective:*** Prospective memory (PM) is the ability to remember to carry out an intended action. Working memory is the ability to store information in mind while processing potentially distracting information. The few previous studies of PM in autism spectrum disorder (ASD) have yielded inconsistent findings. Studies of working memory ability in ASD have suggested a selective impairment of “visual working memory.” However, it remains unclear whether any such impairment is the result of diminished (domain-specific; visual/verbal) storage capacity or diminished (domain-general) processing capacity. We aim to clarify these issues and explore the relation between PM and working memory in ASD. ***Method:*** Seventeen adults with ASD and 17 age- and IQ-matched comparison participants completed experimental measures of both event-based (perform action *x* when event *y* occurs) and time-based (perform action *a* at time *b*) PM, plus a self-report measure of PM skills. Participants also completed a working memory test battery. ***Results:*** Participants with ASD self-reported diminished PM skill, and showed diminished performance on the time-based, but not event-based, PM task. On the working memory test battery, visual but not verbal storage capacity was diminished among participants with ASD, as was processing ability. Whereas visual storage was associated with event-based PM task performance among comparison participants, verbal storage was associated among ASD participants. ***Conclusions:*** ASD appears to involve a selective deficit in time-based PM and a selective difficulty with aspects of working memory that depend on the storage of visual information. However, event-based PM may be achieved through compensatory strategies in ASD.

Autism spectrum disorder (ASD) is a developmental disorder diagnosed on the basis of significant behavioral impairments in social interaction, communication, and behavioral flexibility (American Psychiatric Association, 2000; World Health Organization, 1993). Although a wealth of research has explored the nature of retrospective memory (i.e., memories or forms of learning acquired from past experiences) in ASD, very little research has been conducted in ASD on a form of memory that is focused on the future and is vital for flexible everyday living, namely *prospective* memory (PM). PM is the ability to remember to carry out an intended action after a delay without any explicit instruction to do so (Kliegel, McDaniel, & Einstein, 2008). Common examples of PM include remembering to give someone a message, remembering to pay a bill on time, or remembering to keep an appointment.

A critical distinction is drawn between two types of PM (Einstein & McDaniel, 1990; Einstein, Richardson, Guynn, Cunfer, & McDaniel, 1995; Harris & Wilkins, 1982). On the one hand, event-based PM is the ability to remember to carry out an intention upon the occurrence of a particular, prespecified *event* (e.g., remembering to remove a pan from the stove *when the timer goes off*). On the other hand, time-based PM involves remembering to execute an intention at a particular time point (e.g., remembering to remove a pan from the stove *in 10 minutes’ time*).

Event-based PM is thought to require fewer executive resources than time-based PM, because the occurrence of the key event (e.g., the timer going off) can serve as a *cue*, which automatically reactivates one’s prior intention. Time-based PM appears to rely much more heavily on internal control mechanisms and the *self-initiated* reactivation of one’s intention, given that no external cues are available (e.g., Einstein et al., 1995).

Research on PM among older people has revealed a consistent pattern of decline with age. In a review of the literature, Henry, MacLeod, Phillips, and Crawford (2004) concluded that, in laboratory settings, younger adults tend to show superior event- and time-based PM relative to older adults. However, performance differences between younger and older adults are significantly larger on time-based than event-based tasks. Given that time-based tasks entail a high executive load, this pattern of performance may be attributable to loss of frontal lobe tissue and an associated decline in executive control seen in normal aging (Raz, 2000). This pattern of age-related decline in PM among older people is mirrored by improvements with age among typically developing children, especially on time-based tasks, which may reflect maturation of the frontal lobes (e.g., Kerns, 2000; Ward, Shum, McKinlay, Baker-Tweney, & Wallace, 2005). This research is potentially highly relevant to our understanding of memory in ASD; memory impairments in ASD have been compared with those seen in older individuals from the typical population, and the disorder is known to involve deficits in some aspects of executive functioning (see Kenworthy, Yerys, Anthony, & Wallace, 2008).

## PM in ASD

To our knowledge, only two studies have explored both time-based and event-based PM among the same sample of individuals with ASD. Altgassen, Koban, and Kliegel (2012) assessed a sample of intellectually high-functioning adults with ASD, and an age- and IQ-matched neurotypical comparison group, using a naturalistic ongoing task (preparing breakfast) in which two event-based and two time-based PM tasks were embedded. In this naturalistic task, participants with ASD carried out both the event-based and time-based PM instructions significantly less frequently than comparison participants. Moreover, participants with ASD also performed significantly less well than comparison participants on a more traditional laboratory (i.e., “pencil-and-paper”) test of event-based PM. Taken together, these findings suggest an across-the-board difficulty with PM in ASD. However, there are reasons for caution in interpreting these findings. In particular, participants with ASD performed markedly less well than comparison participants on the ongoing naturalistic task, completing significantly fewer of the predefined ongoing tasks, adhering less frequently to predefined rules, monitoring time less frequently, and showing less efficiency overall in carrying out their plans. This suggests a pervasive difficulty with completing the task as a whole,^1^ and raises the question of whether participants with ASD fully encoded and stored the task instructions. This question is particularly pertinent, given that a posttask assessment of participants’ memory for task instructions, as is usually conducted in studies of PM, was apparently not carried out in Altgassen et al.’s study. Moreover, there is an inherent danger with completing both event- and time-based PM tasks within the same block of the ongoing task; if only one form/aspect of PM is impaired, then difficulties with this could impact negatively on the other aspect that would not, under normal circumstances, be diminished.

In Williams, Boucher, Lind, and Jarrold (2013), intellectually high-functioning children with ASD, plus age- and IQ-matched comparison participants, completed both an event-based task and a time-based PM task. The ongoing task had the same structure for both versions, but the event-based and time-based PM components were assessed in separate blocks (unlike in the Altgassen et al., 2012, study). Crucially, participant groups were matched for ongoing task performance and all participants showed full retrospective memory for task instructions. Thus, any difficulties with the experimental tasks among participants with ASD could not be a secondary consequence of difficulties with extraneous, non-PM task factors. The experimental results showed that participants with ASD had significantly *more* time-based PM failures than comparison participants, despite making nonsignificantly *fewer* omissions on the event-based task. Williams, Boucher, et al.’s finding that event-based PM was unimpaired in ASD was in keeping with the findings of one other study that explored event-based PM only (Altgassen, Schmitz-Huebsch, & Kliegel, 2010; see also Jones et al., 2011, but see Brandimonte, Filippello, Coluccia, Algassen, & Kliegel, 2011).

However, an immediate difficulty with interpreting the findings of unimpaired behavioral performance on measures of event-based PM is that individuals with ASD may have employed atypical, compensatory strategies in order to *perform* well despite limited underlying *competence*. A common approach to intention completion among neurotypical individuals is to encode and store one’s intention to act in the future (e.g., to buy milk on the way home from work), to then cease focusing attention on the intention while getting on with one’s other activities (e.g., work), and then to spontaneously retrieve the intention at the appropriate point (e.g., as one approaches the supermarket on one’s walk home). This kind of “spontaneous retrieval” route to PM is self-reported by over 70% of neurotypical adults and children (e.g., Einstein & McDaniel, 1996; Ward et al., 2005), although nonautomatic processing may nonetheless be required for successful PM in some (particularly demanding) circumstances (see Einstein & McDaniel, 1996). However, in the studies by Williams, Boucher, et al. (2013) and Altgassen et al. (2010), individuals with ASD may have succeeded on the event-based PM tasks merely by *rehearsing* their intention (in working memory) throughout the task. Under these circumstances, successful PM performance would (by definition) depend almost entirely on working memory, which is defined as the ability to hold information in mind (in this case, one’s intention) in the face of potentially distracting activity (in this case, one’s ongoing activities; Baddeley, 1986; Jarrold & Towse, 2006). Note that such a strategy would result in a greater chance of PM success in an event-based task than in a time-based task (cf. Williams, Boucher, et al., 2013), because only in the former is there a cue (i.e., the event itself) to tell you when to act. Without such a cue, one may simply miss the appropriate point to act (i.e., to switch activities) even though one is constantly rehearsing one’s intention.

A logical consequence of the hypothesis that individuals with ASD are atypical in relying on verbal rehearsal of task instructions to succeed on PM tasks is that verbal working memory is itself relatively unimpaired in this disorder. A thorough review of studies of working memory ability in ASD concluded that there is robust evidence of deficits on spatial/visual working memory tasks, whereas the evidence for diminished performance on verbal working memory tasks is negligible (Kenworthy et al., 2008). Thus, the notion that verbal working memory is intact in ASD is perfectly in keeping with the possibility that individuals with this disorder employ verbally based strategies on PM tasks (as they do on some retrospective memory tasks; e.g., Williams, Bowler, & Jarrold, 2012). However, there are several issues to bear in mind when considering working memory ability in ASD.

A key distinction in the cognitive psychology literature is between short-term and working memory, with the former referring to individuals’ ability to *store* or hold in mind information in correct serial order, and the latter referring to specific situations in which the storage of information has to take place *in the face of concurrent distracting processing* (see Jarrold & Towse, 2006). Thus, whereas short-term memory (STM) tasks require only storage, and involve participants simply recalling a sequence of just-presented items in correct serial order (e.g., digit span, Corsi block span), working memory tasks involve both processing *and* storage of information (Daneman & Carpenter, 1980). Consequently, one of the most commonly used and widely accepted measures of working memory is the complex span procedure (Conway et al., 2005), in which the presentation of each successive storage item is paired with a period of potentially distracting processing activity; again, serial recall of the set of storage items is required. Although STM and working memory measures therefore overlap to some degree in typical development, they are consistently distinguishable in factor analyses (Alloway, Gathercole, & Pickering, 2006; Bayliss, Jarrold, Baddeley, Gunn, & Leigh, 2005; Bayliss, Jarrold, Gunn, & Baddeley, 2003; Gathercole, Pickering, Ambridge, & Wearing, 2004; Kane, Hambrick, & Conway, 2005). As such, any potential impairment of working memory in ASD could be due to either diminished storage capacity, diminished processing ability, or a reduction in the executive capacity required to coordinate storage and processing (Bayliss et al., 2003). At present, it is not clear from the existing literature what the basis of any potential diminution of working memory is in ASD.

A further issue that is essential to bear in mind concerns the domain-specificity/domain-generality of working memory. Current theories of the relation between short-term and working memory in both neurotypical children and adults suggest that the short-term *storage* of to-be-remembered information rests on the functioning of domain-*specific* systems, in that verbal and visuospatial STM can be dissociated experimentally and clinically (e.g., Hale, Myerson, Rhee, Weiss, & Abrams, 1996; Jarrold, Baddeley, & Hewes, 1999). In contrast, *processing* of information in working memory tasks appears to depend on a domain-*general* ability that is not influenced by the modality (visual/verbal) of the material-to-be-processed (Bayliss et al., 2003; Kane et al., 2005). As such, the situation is not straightforward enough to claim that visual (or verbal) working memory is (specifically) impaired in ASD, as some have argued (e.g., Kenworthy et al., 2008), because the matter is conceptually more complex than this. It may be that visual STM is impaired in ASD, and that this contributes to a poor performance on working memory tasks that involve the storage of visual information, without there being any fundamental difficulty in combining storage and processing operations in working memory in general in ASD.

In the current study, we were able to explore these issues in ASD by employing a version of the test battery that Bayliss et al. (2003, 2005) developed to assess the relative contributions of processing and storage abilities to complex span among neurotypical adults and children. In Bayliss et al.’s (2003) study, either a verbal or visual processing task was combined with verbal or visual storage demands to create four complex working memory span tasks. Crucially, participants also completed separate tests of these exact visual and verbal storage, and processing requirements, which were measured independently. This allowed Bayliss et al. to decompose complex span performance into its storage and processing components, as well as any residual (potentially executive) variation. This general approach was adopted here to enable us to examine the extent to which storage capacity, processing efficiency, and the ability to combine storage and processing are impaired, or otherwise, in ASD. However, because previous work has indicated that processing efficiency depends on domain-general resources, the current study only included one type of processing, namely, verbal processing. However, storage modality was varied (verbal vs. visual) to create two complex span tasks.

In the current study, participants completed experimental measures of both event-based and time-based PM, as well as completing a self-report measure of both prospective and retrospective memory abilities. Participants also completed the modified Bayliss et al. (2003) working memory test battery just described. The aims of the study were threefold.

First, we aimed to investigate the extent to which PM is impaired in ASD. On the self-report measure (for full details of the measure, see Method section), we predicted that participants would report everyday difficulties with PM (as well as with retrospective memory). In terms of experimental task performance, we predicted that significantly more PM failures (i.e., failures to carry out the PM instruction at the appropriate point) would be observed among ASD participants than among comparison participants on the time-based experimental task but not on the event-based experimental task. Moreover, on the time-based task, we predicted that even when participants with ASD *did* successfully remember to carry out the PM instruction in the target window of time (which was ±8 s from the target time; see Method section), they would do so at a time that was closer to the end of this window (i.e., more distant from the actual target time) than would comparison participants. In other words, even when participants with ASD were successful, they would be less *precise* than comparison participants at carrying out the intended action. With regard to event-based task performance, however, we predicted that there would *not* be a significant group difference in the distance from the target event at which participants carried out the PM instruction. That is, we predicted that participants with ASD would not be less precise than comparison participants on successful PM trials of the event-based task.

Second, we aimed to clarify the profile of strengths and difficulties in working memory among individuals with ASD. With regard to performance on the simple span tasks, we predicted that visuospatial storage capacity would be diminished, whereas verbal storage capacity would be undiminished. We were uncertain whether processing ability would be diminished among participants with ASD. With regard to the complex span tasks, several possibilities were evident. One possibility was that executive difficulties with combining storage and processing requirements among participants with ASD would lead to impairments in all forms of complex span, regardless of whether the visual or verbal information is involved. An alternative possibility was that predicted difficulties with visuospatial storage would lead to a diminution of performance on the complex span task involving visual information only.

Third, we aimed to explore the link between PM and working memory. Here, we had the specific prediction that there would be a unique relation between verbal storage capacity and event-based PM among individuals with ASD.

Finally, we explored the link between self-reported memory ability and experimental PM task performance. This allowed us to explore the extent to which individuals with ASD are self-aware of their own PM abilities; accurate self-awareness would be implied if there was a significant correlation between self-reported memory ability and experimental PM task performance. It should be noted that this exploration was conducted as a result of comments provided by an anonymous reviewer of the original draft of this article. Thus, analyses concerning this aim are post hoc and exploratory.

## Method

### Participants

Seventeen adults with ASD (14 male) and 17 neurotypical comparison adults (14 male) took part in this experiment, after providing written informed consent to take part. Participants in the ASD group had all received formal diagnoses of autistic disorder (*n* = 4) or Asperger’s disorder (*n* = 13), according to conventional criteria (American Psychiatric Association, 2000; World Health Organization, 1993). All participants with ASD completed the Autism Spectrum Quotient (AQ; Baron-Cohen, Wheelwright, Skinner, Martin, & Clubley, 2001), a self-report measure of ASD features, and 13 of 17 were administered the Autism Diagnostic Observation Schedule (ADOS; Lord et al., 2000), a detailed observational assessment of ASD features. Four participants with ASD did not consent to complete the ADOS because they did not wish to be filmed. All comparison participants completed the AQ.

All participants who completed the ADOS scored above the defined cutoff for ASD (total score ≥7; Lord et al., 2000). The mean ADOS total score of the ASD group was in the autism range. Fifteen of the 17 participants with ASD scored above the defined cutoff for ASD on the AQ (total score ≥26; Woodbury-Smith, Robinson, Wheelwright, & Baron-Cohen, 2005). The two participants with ASD who scored below 26 on the AQ each scored well above the defined ASD cutoff on the ADOS (each participant scored 12). The four participants with ASD who did not complete the ADOS all scored well above the defined ASD cutoff on the AQ (all scores >35). All participants in the comparison group scored below the defined cutoff for ASD on the AQ. No participant in either group reported any current use of psychotropic medication or illegal recreational drugs, and none reported any history of neurological or psychiatric illness other than ASD.

Using the Wechsler Abbreviated Scale of Intelligence (WASI; Wechsler, 1999), the groups were equated closely for verbal and nonverbal ability. The groups were also equated closely for chronological age. Participant characteristics are presented in Table 1.[Table-anchor tbl1]

Ethical approval for this study was obtained from Durham University Ethics Committee.

### Tests and Procedures

#### Self-reported prospective and retrospective memory ability

All participants completed the Prospective and Retrospective Memory Questionnaire (PRMQ; Smith et al., 2000). The PRMQ is a 16-item self-report measure of everyday slips in prospective and retrospective memory. Each question is on a scale of 0 to 5, where 0 corresponds to *never having a problem* and 5 corresponds to *very often having a problem*. Eight items concern PM difficulties (e.g., “Do you decide to do something in a few minutes’ time and then forget to do it?”) and eight items concern retrospective memory difficulties (e.g., “Do you fail to recall things that have happened to you in the last few days?”).

Factor analytic studies among the typical population reveal a tripartite structure of the PRMQ, including a general memory factor, plus orthogonal factors capturing retrospective memory and PM abilities, respectively (e.g., Crawford, Henry, Ward, & Blake 2006; Crawford, Smith, Maylor, Della Sala, & Logie, 2003; Rönnlund, Mäntylä, & Nilsson, 2008).

#### PM task

**Figure 1 fig1:**
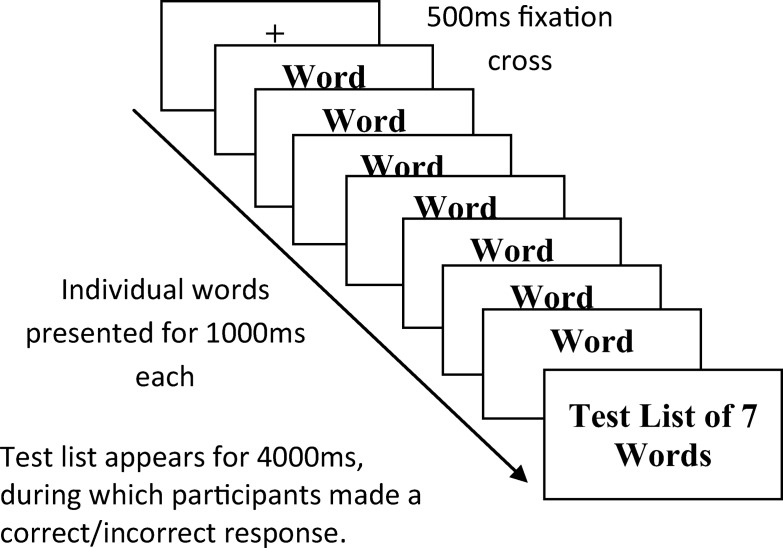
Structure of a trial of the ongoing task.

The ongoing task (a trial of which is illustrated in Figure 1), in which the PM tasks/instructions were embedded, took the same format for both the event-based and time-based PM conditions. In each condition, the ongoing task lasted for 10 min and was presented on a ThinkPad laptop. Participants were presented with a series of study lists (80 in total across both conditions) that consisted of seven individually presented high-frequency words shown at a rate of one word per second. After the presentation of each study list, there appeared a test list that contained seven words on a single screen. The test list remained on screen for 4 s. Participants were asked to make a yes–no recognition judgment, via keyboard button press, about whether the words in the test list were the same as the words in the immediately preceding study list. On 50% of trials, the words in the test list were identical to those in the study list. In the remaining 50% of trials, the test list differed from the study list by one item. The lure item in these incongruent trials was placed in random position within the test list. Participants were awarded a score of one for every trial in which they made a correct recognition judgment and a score of zero for every trial in which they made an incorrect recognition judgment. In each PM condition, there were a total of 40 ongoing trials, thus yielding an ongoing task score of between 0 and 40. At the end of each trial, a blank screen appeared for 1.5 s, followed by a 500-ms fixation cross to alert the participant that the next trial was about to begin.[Fig-anchor fig1]

Two sets of 280 high-frequency words were employed as stimuli for the ongoing task. The use of each set was counterbalanced across the time-based and event-based PM conditions. The sets were equated for mean syllable length of items, and for mean word frequency, as indexed by Kucera and Francis (1967) and reported in the MRC Psycholinguistic Database (Coltheart, 1981). Confirming the adequacy of this matching, a multivariate analysis of these measures across the two sets revealed a nonsignificant main effect of set using Wilks’ criterion, *F*(2, 557) = 0.93, *p* = .39.

Verbal instructions for both the ongoing task and for the PM component of the task in each condition were given by the experimenter immediately prior to testing. Participants completed four practice trials of the ongoing task before being given the PM instructions.

In the time-based condition, participants were told that they should press the “P” key at 2-min intervals throughout the ongoing task (i.e., at Minutes 2, 4, 6, 8, and 10). They were informed that they could bring up a digital clock at any time throughout the ongoing task by pressing the spacebar. The clock began running at the time the ongoing task was started, so displayed the exact task duration in minutes and seconds up to that point. After the spacebar had been pressed, the clock remained on screen for 1.5 s before disappearing. A PM failure in this condition was defined as a failure to press the P key within a 16-s window around each 2-min target time (i.e., within 8 s prior to or 8 s after each 2-min mark). For example, an individual who pressed the P key at 2 min 9 s would be considered to have had a PM failure. This time frame was selected to ensure that even if the 2-min target time fell during the middle of an ongoing trial (i.e., during the presentation of items in the study phase of the ongoing task), participants would still have time to make a PM response within the target window. In other words, even if the 2-min target time fell during the study phase of an ongoing trial, participants would still have time to make a PM response within the target window if they waited until after the study phase to press the P key. For example, if a 4-min interval fell during an ongoing trial on which the first stimulus word had just been presented, 8 s allows for participants to learn all the stimuli in the ongoing trial and still make a successful PM response if they press the P key at the end of the ongoing trial.

In the time-based condition, measures recorded included proportion of PM failures (number of times participants failed to press the P key within the predetermined response window divided by the number of PM trials; 0 to 1); ongoing task score (total number of trials in which participants made the correct recognition judgment; 0 to 40); and the number and distribution of clock checks throughout the task. Breaking down trials into intervals to measure the distribution of clock checks is typical in studies of PM, with an increase in checking toward the end of the target period being thought to reflect strategic monitoring for the target time (e.g., Kerns, 2000; Mäntyla, Carelli, & Forman, 2007; Mackinlay, Kliegel, & Mäntyla, 2009). Finally, across all *successful* PM trials (i.e., trials on which the P key was pressed within 8 s on either side of each 2-min target time), we also measured the mean temporal distance from the 2-min target that participants carried out the PM instruction.

In the event-based condition, the PM instruction was to press the “M” key every time a musical instrument appeared in the *test* list of the ongoing task. Specifically, participants were told that if a musical instrument appeared in the test list, then they should press the M key instead of making a yes–no recognition judgment (i.e., they should ignore the ongoing trial and respond to the target event). One of four musical instruments (piano, guitar, drum, or violin) appeared in the 8th test list of the task and then every 10 trials thereafter (i.e., Trials 8, 18, 28, and 38). Musical instruments did not appear in any *study* list. A PM failure in this condition was defined as a failure to press the M key during the presentation of a test list that contained a musical instrument). Measures recorded were proportion of PM failures (number of times participants failed to press the M key after the presentation of a musical instrument divided by the number of PM trials; 0 to 1) and ongoing task score (total number of trials in which participants made the correct recognition judgment; 0 to 40). With regard to the ongoing task score, on PM target trials in which a musical instrument appeared in the test list, participants were given a score of 1 for the ongoing task if they pressed the M key. This is because, by definition, if participants responded to the musical instrument, then they must have recognized that the test and study lists were incongruent with one another (musical instruments never appeared in study lists). If they did *not* press the M key on target trials (i.e., if they had a PM failure), then they were given a score of 1 for the ongoing task if they correctly recognized that the test list did not contain the same words as the study list (i.e., made a “no” judgment) or a score of 0 if they incorrectly judged that the test list did contain the same words as the study list (i.e., made a “yes” judgment). As such, ongoing task performance was independent of PM task performance. Finally, across all *successful* PM trials, we also measured the time taken to carry out the PM instruction after the appearance of the relevant test list.

At the end of each PM task, retrospective memory for PM instructions was assessed. All participants were able to recall the PM instructions, including necessary key presses, in both conditions. The order in which the event-based and time-based PM tasks were completed was counterbalanced across participants.

#### Working memory task battery

We employed a version of the working memory test battery devised and used by Bayliss et al. (2003, 2005).

##### Complex span tasks

We employed two complex span tasks that were formed by crossing verbal processing with each of the two types of storage (verbal and visual). In each task, participants were presented with a series of screens that each showed nine different colored squares spaced in a random arrangement on a gray background. Each square contained one of the numbers 1 to 9, and the position of these numbers changed on each screen. The verbal processing element involved the participant hearing an auditorily presented item name (e.g., “banana”) and being required to find and click the mouse cursor on the correspondingly colored circle (i.e., yellow) as quickly as possible. Once a target square had been selected, all other squares disappeared, and the selected square remained on the screen until a total of 4 s had elapsed since the onset of auditorily presented object name. The next processing episode began immediately after this 4-s period. If participants failed to click on a square within 3 s of the presentation of the object name, the correct square appeared individually at this point and remained on screen for the remaining 1 s of the processing window. There was a 250-ms window, in which a blank screen was shown, between each processing episode. Object names were chosen from a set that had previously been shown to reliably indicate the target color (Bayliss et al., 2003). The names were prerecorded by a male experimenter and played through computer speakers during the tasks.

The participant’s task then depended on the type of storage required. In tasks involving verbal storage, participants had to remember the digit shown in the square that they had clicked on, whereas in tasks involving visuospatial storage, they remembered the spatial position the square appeared in (this contrast between serial recall of digits as opposed to spatial locations is one that is commonly used to tap verbal as opposed to visuospatial storage in the working memory literature; e.g., Alloway et al., 2006; Hale, Bronik, & Fry, 1997). At the end of the trial, participants were required to recall this information in correct serial order. In the tasks involving verbal storage, participants had to repeat the digits that had been presented in the previously located target squares, and in the visual storage conditions, they were shown a response screen that displayed all nine of the potential target locations and had to click the cursor on the subset of locations occupied by the previously identified target squares in the order in which those target locations had originally been presented. In this way, we generated two directly comparable tasks from a single display: verbal processing with verbal storage, and verbal processing with visuospatial storage. In each task, trials increased in length from three to eight sequences. There were three trials at each sequence length. Each trial was considered to have been successfully completed if all items were recalled in correct order. If at least one of the three trials at a given sequence length was successfully completed, the participant was given another set of (three) trials at a greater sequence length. When none of the trials at a given sequence length was successfully completed (or when the participant completed Level 6, which involved eight-item sequences), the participant moved on to the next task. For both visual and verbal complex span tasks, participants completed two practice trials before experimental trials began.

Participants’ complex span was determined by averaging the number of items in the last three trials that were recalled in correct serial order. Thus, an individual who recalled one (out of three) six-item trials and two (out of three) five-item trials would have a span score of 5.33 (that is, [5 + 5 + 6]/3).

##### Processing efficiency task

A separate verbal processing task was completed by all participants. This processing task was identical to that involved in the complex span tasks but did not involve any storage requirement. Again, participants were presented with a series of screens, each of which showed nine different colored squares. For each screen, participants heard an auditorily presented item name and were required to find and click the mouse cursor on the correspondingly colored square as quickly as possible. Once the participant made their response, however long it took, the next trial was presented. In total, participants completed 40 processing-only trials, although the first four trials were considered practice trials and hence excluded from later analysis. Across the 36 test trials, the average time participants took to select the correct colored square was considered as the measure of processing efficiency/speed. Only trials on which participants selected the correct square were included in this measure.

##### Storage tasks

All participants also completed separate tasks assessing verbal and visual storage capacity, respectively. These storage tasks were structurally identical to those in the complex span tasks but did not involve any additional processing requirement. Storage span was calculated in the same way that complex span was. In both storage tasks, nine blank squares initially appeared on the screen in the same apparently random arrangement as used in all the tasks. One square flashed up with a number between one and nine contained inside it. This square remained highlighted for 1.4 s before going blank. After a 250-ms blank interval, a different square containing another number flashed up. Participants were presented with sequences ranging from three to eight squares. In the verbal storage task, after presentation of the last item in each trial, the screen went blank and the participant was invited to recall, in serial order, the digits contained in the previously highlighted squares. In the visual storage condition, after presentation of the last item in each trial, participants were shown a response screen that displayed all eight of the potential target locations. Just as in the complex visual span task, participants had to click the cursor on the subset of locations of occupied by the previously identified target squares in the order in which those target locations had originally been presented. There were three trials at each sequence length. Participants completed two practice trials before experimental trials began.

Across all participants, the presentation of tasks was fixed in the sense that complex span tasks were always completed first, followed by the storage-only tasks, followed by the processing efficiency-only task. However, task modality was counterbalanced across participants. Half of the participants from each group completed the verbal versions of the complex span and storage-only tasks first, whereas the other half completed the visual versions first. Hence, half of participants completed tasks in the following order: verbal complex span, visual complex span, verbal storage only, visual storage only, processing efficiency. The remaining half of participants completed tasks in the following order: visual complex span, verbal complex span, visual storage only, verbal storage only, processing efficiency.

## Results

### PM Data

#### Do participants with ASD self-report PM difficulties in everyday life?

Table 2 shows the mean raw scores and *t* scores among ASD and comparison participants on each of the PM and retrospective memory subscales of the PRMQ. A mixed ANOVA was conducted on the raw scores from each subscale, with group (ASD/comparison) as the between-participants variable and subscale (prospective/retrospective) as the within-participants variable. The main effect of subscale was significant, *F*(1, 31) = 18.98, *p* < .001, partial η^2^ = .38 reflecting higher raw scores on the Prospective Memory subscale than the Retrospective Memory subscale. The main effect of group was also significant, *F*(1, 31) = 9.25, *p* = .005, partial η^2^ = .23, reflecting the superior performance of comparison participants overall. Finally, the interaction between group and subscale was not significant, *F*(1, 31) = 0.39, *p* = .54, partial η^2^ = .01.[Table-anchor tbl2]

Among comparison participants, the standardized (*t*) score on the Prospective Memory subscale was not significantly different from the population average of 50, *t*(16) = 1.21, *p* = .24, *d* = 0.28. However, the *t* score on the Retrospective Memory subscale was significantly *above* the population average, *t*(16) = 3.00, *p* = .009, *d* = 0.61. Among participants with ASD, the *t* score on the Prospective Memory subscale was significantly *below* the population average, *t*(15) = 2.20, *p* = .04, *d* = 0.68. The *t* score on the Retrospective Memory subscale was not significantly below the population average, although a (small to modest) trend was observed, *t*(15) = 1.80, *p* = .09, *d* = 0.47.

#### How do participants perform on the ongoing component of the laboratory PM tasks?

Table 3 shows overall ongoing task performance (number of trials, out of 80, on which a correct recognition judgment was made) collapsed across event-based and time-based conditions among ASD and comparison participants. A mixed ANOVA was conducted on these data, with group (ASD/comparison) as the between-participants variable and condition (time-based/event-based) as the within-participants variable. The main effect of condition was not significant, *F*(1, 32) = 0.13, *p* = .72, partial η^2^ = .004. The main effect of group was not significant, *F*(1, 32) = 0.01, *p* = .93, partial η^2^ = .003. Finally, the interaction between group and condition was also not significant, *F*(1, 32) = 0.09, *p* = .77, partial η^2^ < .001. Thus, in terms of ongoing task performance, no difference between the groups approached significance and none were associated with anything other than a negligible/small effect size. As such, any group differences in performance on the PM component of each task cannot be attributed to baseline differences between the groups in ongoing task performance.[Table-anchor tbl3]

#### How do participants perform on the PM component of the experimental tasks?

Table 3 shows the mean proportion of PM failures made by ASD and typically developing (TD) participants in each task (time-based/event-based).^2^ A mixed ANOVA was conducted on these data, with group (ASD/comparison) as the between-participants variable and PM task (time-based/event-based) as the within-participants variable. There was a significant main effect of PM task, *F*(1, 31) = 4.09, *p* = .05, partial η^2^ = .11, reflecting superior performance on the time-based task than in the event-based task. The main effect of group was not significant, *F*(1, 31) = 0.04, *p* = .85, partial η^2^ = .001. However, there was a significant interaction between PM task and group, *F*(1, 31) = 5.87, *p* = .02, partial η^2^ = .16. Independent-samples *t* tests revealed that participants with ASD performed significantly *less well* than comparison participants in the time-based task, *t*(31) = 2.04, *p* = .05, *d* = 0.78, but nonsignificantly *better* than comparison participants in the event-based task, *t*(32) = 1.20, *p* = .24, *d* = 0.43.

Among participants from both groups, performance in both the event-based and time-based tasks was significantly above floor and significantly below ceiling (all *p*s ≥ .04).

#### How precise (i.e., close to the target) were participants’ correct PM actions?

Across successful *time-based* PM trials, participants took, on average, the following number of seconds to carry out the PM instruction after the 2-min target time: ASD, *M* = 2.59, *SD* = 1.15; comparison, *M* = 1.50, *SD* = 0.95.

Across successful *event-based* PM trials, participants took, on average, the following number of seconds to carry out the PM instruction after the appearance of a test list that contained a musical instrument: ASD, *M* = 2.31, *SD* = 0.66; comparison, *M* = 2.01, *SD* = 0.53.

A mixed ANOVA was conducted on these data, with group (ASD/comparison) as the between-participants variable and PM task (time-based/event-based) as the within-participants variable. The main effect of PM task was not significant, *F*(1, 27) = 0.35, *p* = .56, partial η^2^ = .01. The main effect of group was significant, *F*(1, 27) = 9.55, *p* = .005, partial η^2^ = .26, reflecting poorer performance overall among participants with ASD. However, there was a significant interaction between PM task and group, *F*(1, 27) = 5.37, *p* = .03, partial η^2^ = .17. Independent-samples *t* tests revealed that participants with ASD carried out the PM instruction significantly *less* efficiently/precisely than comparison participants in the time-based task, *t*(30) = 2.93, *p* = .007, *d* = 1.04, but not in the event-based task, *t*(29) = 1.40, *p* = .17, *d* = 0.50.

#### How often and at what points did participants check the clock during the time-based PM task?

**Figure 2 fig2:**
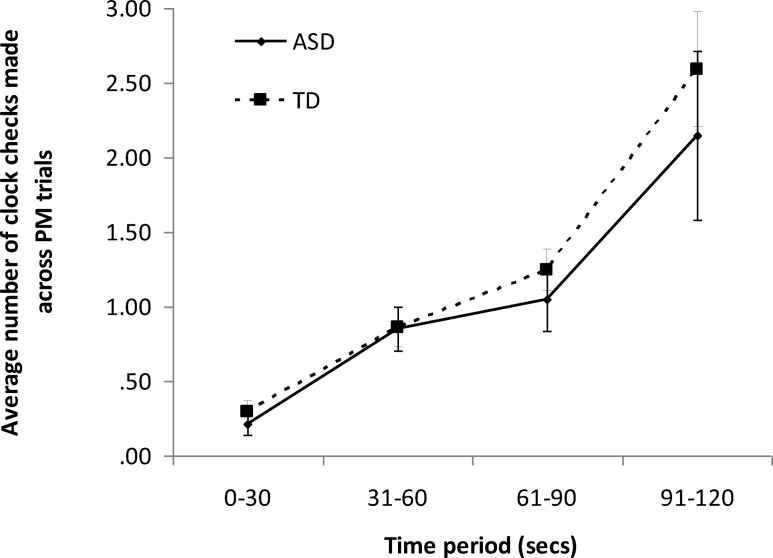
Average number of clock checks (averaged across trials) made by ASD and TD participants during the time-based PM task.

Figure 2 shows the number of clock checks made in each of the four 30-s intervals preceding the target time (averaged across the five time-based PM trials). A mixed ANOVA was conducted on these data, with group (ASD/comparison) as the between-participants variable and time period (0 to 30 s, 31 to 60 s, 61 to 90 s, 91 to 120 s) as the within-participants variable. Mauchly’s test of sphericity was significant, χ^2^(5) = 102.75, *p* < .001. Therefore, Greenhouse-Geisser corrections were employed. There was a significant main effect of time period, *F*(3, 1.16) = 35.42, *p* < .001, partial η^2^ = .53. Within-participant contrasts suggested mainly linear, *F* = 45.80, *p* < .001, and cubic, *F* = 21.45, *p* < .001, effects, although a quadratic effect was also evident to a lesser extent, *F* = 6.23, *p* = .02. The main effect of group was not significant, *F*(1, 31) = 0.45, *p* = .51, partial η^2^ = .01. The interaction between group and time period was also not significant, *F*(3, 1.16) = 0.40, *p* = .56, partial η^2^ = .01. Thus, there was no evidence of any reliable group difference in either the overall frequency of clock checks or the distribution of clock checks across each trial.[Fig-anchor fig2]

#### What was the association between self-reported PM ability on the PRMQ, and event-based and time-based experimental task performance?

Within each group, we explored the extent to which number of PM failures in each experimental task (event-based/time-based) was associated with participants’ raw score on the PM subscale of the PRMQ. In each analysis, we controlled for ongoing task performance in order to gain a pure measure of the relation between PM task performance and self-reported PM ability, independent of the general (non-PM) demands of the experimental PM tasks.

The number of time-based PM failures was not associated significantly with self-reported PM ability among either group, all *r*s ≥ −.11, all *p*s ≥ .67. Among participants with ASD, the number of event-based PM failures was only weakly (negatively) and nonsignificantly associated with raw score on the PM subscale of the PRMQ, *r* = −.06, *p* = .83. However, among comparison participants, the equivalent association was moderate to strong and positive, although not quite statistically significant, *r* = .43, *p* = .08. Thus, among comparison participants, poorer performance on the experimental event-based PM task was associated with poorer self-reported PM ability.

### Working Memory Assessment

#### How efficient were participants at processing information on the processing efficiency task?

In terms of comprehending the meaning of item names, there was no significant difference in the number of trials (out of 36) in which participants with ASD (*M* = 28.71, *SD* = 3.99) and comparison participants (*M* = 30.00, *SD* = 2.83) selected the correct colored square on the independent test of processing efficiency, *t*(32) = 1.09, *p* = .28, *d* = 0.38. Thus, object names had equivalent meanings for ASD and comparison participants. In terms of processing efficiency, however, matters were different. The time (in seconds) that ASD participants took to select the correct square (*M* = 2.30, *SD* = 0.40) was significantly longer than the time taken by comparison participants (*M* = 1.92, *SD* = 0.31), *t*(32) = 3.10, *p* = .004, *d* = 1.07.

#### How large were participants’ complex and simple spans?

Table 4 shows the mean complex and simple spans achieved in verbal and visual modalities by participants from each diagnostic group. These data were subjected to a 2 (group: ASD/comparison) × 2 (task: complex span/simple span) × 2 (modality: verbal/visual) ANOVA. Overall, there were significant main effects of task, *F*(1, 32) = 37.08, *p* < .001; modality, *F*(1, 32) = 30.21, *p* < .001; and group, *F*(1, 32) = 4.75, *p* = .04. These significant main effects reflected the facts that, overall, complex span was smaller than simple span, visual span was smaller than verbal span, and participants with ASD performed less well than comparison participants.[Table-anchor tbl4]

There was a significant interaction between group and modality, *F*(1, 32) = 4.96, *p* = .03. Independent-samples *t* tests revealed that, across the complex span and simple span tasks, participants with ASD performed significantly less well than comparison participants in the visual modality, *t*(32) = 2.86, *p* = .008, *d* = 1.00, but not in the verbal modality, *t*(32) = 0.48, *p* = .64, *d* = 0.17.

No further interactions were significant, all *p*s ≥ .50, all partial η^2^s ≤ .02.

#### What was the association between experimental PM task performance and working memory task performance?

Within each group, we explored the extent to which number of PM failures in each experimental task (event-based/time-based) was associated with participants’ complex span (verbal and visual), simple span (verbal and visual), and verbal processing ability.

In each analysis, we controlled for ongoing task performance in order to gain a pure measure of the relation between PM task performance and working memory, independent of the general (non-PM) demands of the experimental PM task. These partial correlations are shown in Table 5. Among comparison participants, event-based PM performance was associated significantly with simple *visual* span, whereas among ASD participants, event-based PM performance was associated significantly with simple *verbal* span. In this respect, ASD and comparison participants were the mirror image of each other. Among ASD participants, event-based PM performance was additionally significantly associated with verbal processing ability.[Table-anchor tbl5]

## Discussion

Until now, only two studies have investigated both event-based and time-based PM ability among individuals with ASD (Altgassen et al., 2012; Williams, Boucher, et al., 2013). Given that these studies have produced contradictory results, the first aim of the current study was to clarify the profile of PM ability among individuals with this disorder. The second aim of the study was to elucidate the nature of working memory ability among individuals with ASD. In relation to this, it was unclear whether “diminished visual working memory” (as some have claimed characterizes ASD) is the consequence of a reduced capacity to store visual information (simple visual span), lower processing speed, or an executive difficulty with combining storage and processing demands.

In relation to the first aim of the study, we found that individuals with ASD self-reported significant difficulties with PM in their everyday lives. To our knowledge, this is the first study to gain self-reports of PM ability specifically among people with ASD. Altgassen et al. (2012) found self-reported difficulties in executive functions that are thought to be related to PM. While those results are important, they do not establish whether individuals with ASD believe themselves to have actual PM difficulties in everyday life. Our results—from a well-established, reliable, and valid self-report measure of PM ability (the PRMQ)—show that adults with ASD *do* believe themselves to have everyday difficulties with PM, which is important. Interestingly, however, whereas self-reported memory abilities were moderately to strongly associated with experimental event-based PM task performance among comparison participants, there was no such positive association among participants with ASD. This may suggest that self-awareness of PM ability is somewhat more accurate among neurotypical individuals than it is among individuals with ASD, which fits with the view that ASD involves deficits in metacognition (or “theory of own mind”) (see Williams, 2010). However, the analysis that revealed these differences was conducted post hoc and should be treated with caution pending independent replication.

A central finding of the current article was the replication of Williams, Boucher, et al.’s (2013) finding of significantly diminished time-based PM, but undiminished performance event-based PM, among individuals with ASD. Importantly, the effect sizes associated with group differences in PM task performance were very similar in each study. In the Williams, Boucher, et al. study, between-groups differences in time-based and event-based PM task performance were associated with Cohen’s *d* values of 0.68 and 0.17, respectively. The corresponding *d* values in the current study were 0.78 and 0.42. Thus, despite the different sample characteristics in the two studies (children in the Williams, Boucher, et al. study; adults in the current study) and the quite different ongoing task demands of the PM tasks (a driving simulation in the Williams, Boucher, et al. study; a recognition memory task in the current study), the experimental PM results are notably consistent. Among comparison participants, a greater proportion of failures were made on the experimental event-based PM task than on the experimental time-based PM task. Among participants with ASD, the proportion of failures was similar on both tasks. In addition, it is important to note that the failure to observe an event-based PM impairment among participants with ASD is unlikely to be the result of limited statistical power; in both the current study and the Williams, Boucher, et al. (2013) study, participants with ASD showed superior event-based PM than comparison participants, albeit nonsignificantly so. Thus, it is not simply the case that participants with ASD in the current study (or in the study by Williams, Boucher, et al.) had genuinely diminished event-based PM, but that the power of the study was insufficient for this diminution to cross the traditional threshold of statistical significance.

An additional finding in the current study was that even on successful time-based PM trials (i.e., in which participants made the correct PM response within the target response window), participants with ASD were less precise in responding (i.e., responded later within the target window) than comparison participants. Thus, even when participants with ASD did not completely forget to carry out their intention, they nonetheless carried it out at a time that was more distant from the target time than did comparison participants. Arguably, this reduced efficiency has real-world relevance; for example, if one were to take one’s medication consistently later than prescribed, it might have serious detrimental consequences to one’s health.

In relation to the second aim of the study, we found that complex visual span was indeed diminished among individuals with ASD, as other have suggested. The nonsignificant Group × Task and Group × Task × Modality interactions in the omnibus ANOVA indicated complex visual span was no more impaired among individuals with ASD than was simple visual span. Thus, the diminution of complex visual span appeared to be rooted in a reduced capacity to store visual information (simple visual span) but not in a reduced executive capacity to combine storage and processing demands. As a result, our findings confirm previous evidence of a visual storage deficit in ASD (Kenworthy et al., 2008; Williams, Goldstein, Carpenter, & Minshew, 2005) but count against the view that ASD is associated with a general, executively mediated, working memory difficulty. Crucially, there was little evidence that either simple verbal span or complex verbal span were diminished among participants with ASD.

With regard to our finding of undiminished verbal span in ASD, one might argue that the requirement to remember digits, rather than some other form of verbal material, selectively benefited individuals with ASD, given that anecdotal reports and findings of empirical studies have emphasized the relative strength and level of interest in numbers and numerical operations among intellectually high-functioning individuals with ASD (e.g., Baron-Cohen, Richler, Bisarya, Gurunathan, & Wheelwright, 2003). With regard to this possibility, it is important to highlight, on the one hand, evidence showing that neurotypical individuals’ degree of familiarity with the memoranda does influence their performance in tests of verbal STM (see Brener, 1940). Therefore, it is possible that individuals with ASD do particularly well on these verbal storage tasks because of their relatively greater familiarity with digits.

On the other hand, it is important to note that digit span is a commonly used and widely accepted index of verbal STM, and that studies have shown that digit span shares considerable variance with other “verbal” measures such as word span and nonsense-word repetition ability (e.g., Alloway et al., 2006; Gupta, 2003; Kane et al., 2005). Moreover, among individuals with ASD, digit span is associated highly and significantly with nonsense-word repetition ability to the same extent as it is among neurotypical individuals (Williams, Payne, & Marshall, 2013). Thus, in our view, it is unlikely that verbal span was undiminished among participants with ASD in the current study merely because of a hypothetical interest in digits among participants in in this group.

The final aim of the current study was to explore the association between PM ability and working memory ability. We made the specific prediction that verbal storage capacity would be uniquely associated with event-based PM performance among participants with ASD. This prediction was supported by the data. Whereas event-based PM was associated uniquely with simple *visual* span among comparison participants, it was associated uniquely with simple *verbal* span among participants with ASD. Although only correlational in nature, this result is in keeping with the idea that individuals with ASD rely uniquely on verbally mediated strategies to succeed on event-based PM tasks, whereas comparison participants rely more heavily on visually mediated strategies. There was no such correlation between verbal span and *time-based* PM among participants with ASD. However, as discussed, such verbal strategies are less applicable to time-based PM tasks, because there is not a directly perceivable environmental cue to direct participants when to carry out their intention. Thus, even if an individual continuously rehearsed their intention, this would not guarantee that they would succeed in carrying out the intention at the appropriate point. Thus, the lack of a significant correlation between verbal span and time-based PM among participants with ASD was in keeping with our expectations. Of course, as discussed further below, this argument may not be correct. However, it was made a priori and is directly testable.

Notably, among comparison participants, event-based PM was associated with simple visual span rather than simple verbal span. This may provide indirect evidence for a hypothesized link between PM and “episodic future thinking” (Ford, Driscoll, Shum, & Macaulay, 2012). According to this hypothesis, the spontaneous retrieval route, described earlier, is underpinned by the capacity to, at the time of forming one’s intention to act in the future, mentally project oneself into the future to imagine the realization of that intention. Episodic future thinking is fundamentally a visual process that depends on aspects of working memory (e.g., Verfaellie, Race, & Keane, 2012), and thus the relation between visual span and PM in the current study may reflect this link. Neuroimaging studies of PM among the typical population support this possibility and may put the current findings in context. Over a dozen neuroimaging studies have shown that performance on PM tasks, relative to ongoing tasks alone (without PM instructions), is consistently associated with activation in the rostral prefrontal cortex (PFC; Brodmann Area 10; see Burgess, Gonen-Yaacovi, & Volle, 2011). Although activation of the rostral PFC is relatively insensitive to changes in superficial task structure/content (suggesting it relates specifically to a superordinate PM component), results suggest BA10 activation in time-based tasks is more medial than in (laterally activated) event-based tasks (Okuda et al., 2007). Particularly important is the recent finding of Volle et al. (2011) that lesions to right BA10 are associated with specific deficits in time-based, but not event-based, PM. Thus, future neuroimaging studies of PM among individuals with ASD may benefit from a focus on divisions in BA10.

A key issue in the current study is that among neurotypical individuals, PM appears to be related to visuospatial processes, whereas among individuals with ASD, PM is associated with verbal processes. The notion that individuals with ASD employ “compensatory” verbal strategies to succeed on cognitive-experimental tasks is common in ASD research (e.g., Bowler, 1992; Lind & Bowler, 2009; Williams & Happé, 2009). We suggest that this could be the case for event-based PM also. Thus, even though event-based PM task performance was undiminished in the current sample of ASD participants, we suggest that the underlying basis of success may have been different in each group (verbally mediated among ASD participants; visually based among comparison participants). This hypothesis is supported by the different patterns of cognitive correlates among each group of participants. Of course, this kind of evidence is not “water tight” and further research would be necessary to (dis)confirm the hypothesis. For example, the hypothesis could be supported more definitively by assessing the effects on PM task performance of concurrent articulatory suppression (which selectively disrupts verbal mediation; e.g., Murray, 1967) and of concurrent spatial tapping (which selectively disrupts visuospatial thinking; e.g., Hyun & Luck, 2007), respectively. In studies of neurotypical adults, concurrent articulatory suppression does *not* detrimentally affect event-based PM task performance (Marsh & Hicks, 1998; Otani, Landau, Libkuman, St. Louis, & Kazen, 1997), whereas concurrent spatial tapping does negatively influence performance (Marsh & Hicks, 1998). We predict that the opposite pattern would be observed among individuals with ASD.

The current study builds on those few studies that have explored PM in ASD and adds to converging evidence of a selective diminution of time-based PM task performance among individuals with this disorder. The study also provides preliminary evidence of alternative, compensatory strategy use among individuals with ASD to mediate PM. In our view, the systematic study of PM ability in ASD is highly important and long overdue. Of course, individuals with ASD are clearly capable of successful time-based PM under some circumstances; time-based PM was diminished, but certainly not absent, among participants with ASD in the current study. Statistically, the size of this diminution was relatively large (*d* = 0.78). Although it is always wise to question whether statistical significance equates to clinical significance, it is the case that even relatively modest impairments in PM can have serious negative consequences for everyday life and adaptive functioning (e.g., Wilson, 1991). Thus, we believe strongly that more work needs to be done to understand (a) the underlying basis of PM task performance among people with ASD, and (b) the extent to which behavioral difficulties with PM (and its underlying basis) can be successfully remediated among people with ASD. If people with ASD do employ compensatory strategies to succeed on event-based PM tasks, do these adequately support everyday event-based PM? If so, can these strategies be fostered among individuals with the disorder who do not use them in their everyday lives? If the compensation turned out not to adequately support everyday PM, can alternative intervention strategies be implemented? We hope that the current research will motivate and inform future studies that might answer theses questions and, ultimately, enhance the capacity for behavioral flexibility among individuals with ASD.

## Figures and Tables

**Table 1 tbl1:** Participant Characteristics

	Group		*t*	*p*	Cohen’s *d*
	ASD (*n* = 17)	TD (*n* = 17)			
Age	31.06 (9.64)	31.92 (14.17)	0.21	.84	0.07
VIQ	111.41 (15.79)	114.64 (13.95)	0.63	.53	0.21
PIQ	113.47 (15.16)	116.88 (12.23)	0.72	.47	0.25
FSIQ	114.06 (15.16)	117.71 (13.05)	0.75	.49	0.26
AQ total score	35.59 (9.17)	13.24 (6.37)	8.10	< .001	2.78
ADOS total score^a^	12.36 (2.20)	—	—	—	—
*Note.* ASD = autism spectrum disorder; TD = Typically developing; VIQ = verbal intelligence quotient; PIQ = performance intelligence quotient; FSIQ = Full-scale intelligence quotient; AQ = Autism Spectrum Quotient; ADOS = Autism Diagnostic Observation Schedule.					
^a^ Based on 13/17 participants.					

**Table 2 tbl2:** Self-Reported Prospective and Retrospective Memory Ability Among ASD and TD Participants (Means and SDs)

	Group		Cohen’s *d*
	ASD	TD	
Prospective subscale: Raw score	24.69 (8.13)	18.65 (4.60)	0.95
Prospective subscale: *T* score^a^	41.13 (16.14)	52.71 (9.19)	0.95
Retrospective subscale: Raw score	21.00 (5.55)	15.88 (3.60)	1.12
Retrospective subscale: *T* score^a^	45.00 (11.10)	55.24 (7.21)	1.12
*Note.* ASD = autism spectrum disorder; TD = typically developing.			
^a^ *T* scores are standardized to a mean score of 50 (*SD* = 10).			

**Table 3 tbl3:** Time-Based and Event-Based PM Task Performance, and Overall Ongoing Task Performance, Among ASD and TD Participants (Means and SDs)

	Group		*d*
	ASD	TD	
Overall ongoing task score	58.88 (8.87)	58.65 (5.89)	0.03
Proportion of time-based failures	.21 (.32)	.05 (.09)	0.78
Proportion of event-based failures	.19 (.29)	.33 (.36)	0.43
*Note.* PM = prospective memory; ASD = autism spectrum disorder; TD = typically developing.			

**Table 4 tbl4:** Working Memory Task Performance Among ASD and TD Participants (Means and SDs)

	Group		*d*
	ASD	TD	
Complex span: Verbal	6.27 (1.08)	6.41 (0.71)	0.16
Complex span: Visual	4.81 (1.38)	5.92 (0.95)	0.95
Simple span: Verbal	7.14 (1.15)	7.27 (0.76)	0.14
Simple span: Visual	5.72 (1.25)	6.54 (0.82)	0.79
*Note.* ASD = autism spectrum disorder; TD = typically developing.			

**Table 5 tbl5:** Partial Correlations

	Time-based PM failures		Event-based PM failures	
	ASD	Comparison	ASD	Comparison
Complex span: Verbal	−.24	.08	−.36	−.03
Complex span: Visual	.04	.39	−.06	−.30
Storage span: Verbal	−.13	−.38	−.52*	−.08
Storage span: Visual	.04	.06	−.21	−.50*
Verbal processing	.41	−.10	.45*	.06
*Note.* PM = prospective memory; ASD = autism spectrum disorder.				
* *p* < .05.				

## Footnotes

1 In studies of PM, performance on the ongoing task in which the PM targets are embedded needs to be considered when interpreting experimental PM task performance. If participant groups are not equated for ongoing task performance, then group differences in performance on the PM task might result from differences in the ratio of attentional resources devoted to the ongoing task relative to the PM task.

2 Data on the time-based PM task from one participant with ASD was excluded, because of a failure to adhere to task instructions (the participant repeatedly pressed the P key throughout the entire task, despite constant reminders that they should only press the key at the target time).

## References

[c1] Alloway T. P., Gathercole S. E., & Pickering S. J. (2006). Verbal and visuospatial short-term and working memory in children: Are they separable? Child Development, 77, 1698–1716 17107455 10.1111/j.1467-8624.2006.00968.x

[c2] Altgassen M., Koban N., & Kliegel M. (2012). Do adults with autism spectrum disorder compensate in naturalistic prospective memory tasks? Journal of Autism and Developmental Disorders, 42, 2141–2151 22350339 10.1007/s10803-012-1466-3

[c3] Altgassen M., Schmitz-Huebsch M., & Kliegel M. (2010). Event-based prospective memory performance in autism spectrum disorder. Journal of Neurodevelopmental Disorders, 2, 2–8 22127837 10.1007/s11689-009-9030-y PMC3164034

[c4] Altgassen M., Williams T. I., Bölte S., & Kliegel M. (2009). Time-based prospective memory in children with autism spectrum disorder. Brain Impairment, 10, 52–58

[c5] American Psychiatric Association (2000). Diagnostic and statistical manual of mental disorders (4th ed., text rev.), Washington, DC: Author

[c6] Baddeley A. D. (1986). Working memory. Oxford, UK: Oxford University Press

[c7] Baron-Cohen S., Richler J., Bisarya D., Gurunathan N., & Wheelwright S. (2003). The systemizing quotient: An investigation of adults with Asperger syndrome or high-functioning autism, and normal sex differences. Philosophical Transactions of the Royal Society of London: Series B: Biological Sciences, 358, 361–374 10.1098/rstb.2002.1206 PMC1693117 12639333

[c8] Baron-Cohen S., Wheelwright S., Skinner R., Martin J., & Clubley E. (2001). The autism–spectrum quotient (AQ): Evidence from Asperger syndrome/high-functioning autism, males and females, scientists and mathematicians. Journal of Autism and Developmental Disorders, 31, 5–7 11439754 10.1023/a:1005653411471

[c9] Bayliss D. M., Jarrold C., Baddeley A. D., Gunn D. M., & Leigh E. (2005). Mapping the developmental constraints on working memory span performance. Developmental Psychology, 41, 579–597 16060806 10.1037/0012-1649.41.4.579

[c10] Bayliss D. M., Jarrold C., Gunn D. M., & Baddeley A. D. (2003). The complexities of complex span: Explaining individual differences in working memory in children and adults. Journal of Experimental Psychology: General, 132, 71–92 12656298 10.1037/0096-3445.132.1.71

[c59] Bowler D. M. (1992) ‘Theory of mind’ in Asperger’s syndrome. Journal of Child Psychology and Psychiatry, 33, 877–893 1378848 10.1111/j.1469-7610.1992.tb01962.x

[c11] Brandimonte M. A., Filippello P., Coluccia E., Altgassen M., & Kliegel M. (2011). To do or not to do? Prospective memory versus response inhibition in autism spectrum disorder and attention-deficit/hyperactivity disorder. Memory, 19, 56–66 21240748 10.1080/09658211.2010.535657

[c12] Brener R. (1940). An experimental investigation of memory span. Journal of Experimental Psychology, 26, 467–482

[c13] Burgess P. W., Gonen-Yaacovi G., & Volle E. (2011). Functional neuroimaging studies of prospective memory: What have we learnt so far? Neuropsychologia, 49, 2246–2257 21329712 10.1016/j.neuropsychologia.2011.02.014

[c15] Coltheart M. (1981). The MRC psycholinguistic database. Quarterly Journal of Experimental Psychology: A: Human Experimental Psychology, 33, 497–505

[c16] Conway A. R. A., Kane M. J., Bunting M. F., Hambrick D. Z., Wilhelm O., & Engle R. W. (2005). Working memory span tasks: A methodological review and user’s guide. Psychonomic Bulletin and Review, 12, 769–786 16523997 10.3758/bf03196772

[c17] Crawford J. R., Henry J. D., Ward A. L., & Blake J. (2006). The Prospective and Retrospective Memory Questionnaire (PRMQ): Latent structure, normative data, and discrepancy analysis for proxy-ratings. British Journal of Clinical Psychology, 45, 83–104 16480568 10.1348/014466505X28748

[c18] Crawford J. R., Smith G., Maylor E. A., Della Sala S., & Logie R. H. (2003). The Prospective and Retrospective Memory Questionnaire (PRMQ): Normative data and latent structure in a large non-clinical sample. Memory, 11, 261–275 12908675 10.1080/09658210244000027

[c19] Daneman M., & Carpenter P. A. (1980). Individual differences in working memory and reading. Journal of Verbal Learning and Verbal Behavior, 19, 450–466

[c20] Einstein G. O., & McDaniel M. A. (1990). Normal aging and prospective memory. Journal of Experimental Psychology: Learning, Memory, and Cognition, 16, 717–726 10.1037//0278-7393.16.4.717 2142956

[c21] Einstein G. O., & McDaniel M. A. (1996). Retrieval processes in prospective memory: Theoretical approaches and some new empirical findings In Brandimonte M., Einstein G. O., & McDaniel M. A. (Eds.), Prospective memory: Theory and applications (pp. 115–141). Hillsdale, NJ: Erlbaum

[c22] Einstein G. O., Richardson S. L., Guynn M. J., Cunfer A. R., & McDaniel M. A. (1995). Aging and prospective memory: Examining the influences of self-initiated retrieval-processes. Journal of Experimental Psychology: Learning, Memory, and Cognition, 21, 996–1007 10.1037//0278-7393.21.4.996 7673871

[c23] Ford R. M., Driscoll T., Shum D., & Macaulay C. E. (2012). Executive and theory-of-mind contributions to event-based prospective memory in children: Exploring the self-projection hypothesis. Journal of Experimental Child Psychology, 111, 468–489 22169353 10.1016/j.jecp.2011.10.006

[c24] Gathercole S. E., Pickering S. J., Ambridge B., & Wearing H. (2004). The structure of working memory from 4 to 15 years of age. Developmental Psychology, 40, 177–190 14979759 10.1037/0012-1649.40.2.177

[c25] Gupta P. (2003). Examining the relationship between word learning, nonword repetition, and immediate serial recall in adults. Quarterly Journal of Experimental Psychology: A: Human Experimental Psychology, 56, 1213–1236 10.1080/02724980343000071 12959911

[c26] Hale S., Bronik M. D., & Fry A. F. (1997). Verbal and spatial working memory in school-aged children: Developmental differences in susceptibility to interference. Developmental Psychology, 33, 364–371 9147843 10.1037//0012-1649.33.2.364

[c27] Hale S., Myerson J., Rhee S. H., Weiss C. S., & Abrams R. A. (1996). Selective interference with the maintenance of location information in working memory. Neuropsychology, 10, 225–240

[c28] Harris J. E., & Wilkins A. J. (1982). Remembering to do things: A theoretical framework and an illustrative experiment. Human Learning, 1, 123–136

[c30] Henry J. D., MacLeod M. S., Phillips L. H., & Crawford J. R. (2004). A meta-analytic review of prospective memory and aging. Psychology and Aging, 19, 27–39 15065929 10.1037/0882-7974.19.1.27

[c32] Hyun J. S., & Luck S. L. (2007). Visual working memory as the substrate for mental rotation. Psychonomic Bulletin & Review, 14, 154–158 17546746 10.3758/bf03194043

[c33] Jarrold C., Baddeley A. D., & Hewes A. K. (1999). Genetically dissociated components of working memory: Evidence from Down’s and Williams syndrome. Neuropsychologia, 37, 637–651 10390025 10.1016/s0028-3932(98)00128-6

[c34] Jarrold C., & Towse J. T. N. (2006). Individual differences in working memory. Neuroscience, 139, 39–50 16325344 10.1016/j.neuroscience.2005.07.002

[c35] Jones C. R. G., Happé F., Pickles A., Marsden A. J. S., Tregay J., Baird G., . . . Charman T. (2011). “Everyday memory” impairments in autism spectrum disorders. Journal of Autism and Developmental Disorders, 41, 455–464 20635196 10.1007/s10803-010-1067-y

[c36] Kane M. J., Hambrick D. Z., & Conway A. R. A. (2005). Working memory capacity and fluid intelligence are strongly related constructs: Comment on Ackerman, Beier, and Boyle (2005). Psychological Bulletin, 131, 66–71 15631552 10.1037/0033-2909.131.1.66

[c37] Kenworthy L., Yerys B. E., Anthony L. G., & Wallace G. L. (2008). Understanding executive control in autism spectrum disorders in the lab and in the real world. Neuropsychology Review, 18, 320–338 18956239 10.1007/s11065-008-9077-7 PMC2856078

[c38] Kerns K. A. (2000). The CyberCruiser: An investigation of development of prospective memory in children. Journal of the International Neuropsychological Society, 6, 62–70 10761368 10.1017/s1355617700611074

[c39] Kliegel M., McDaniel M. A., & Einstein G. O. (2008). Prospective memory: Cognitive, neuroscience, developmental, and applied perspectives. Mahwah, NJ: Erlbaum

[c40] Kucera H., & Francis W. N. (1967). Computational analysis of present day American English. Providence, RI: Brown University Press

[c41] Lind S. E., & Bowler D. M. (2009). Language and theory of mind in autism spectrum disorder: The relationship between complement syntax and false belief task performance. Journal of Autism and Developmental Disorders, 39, 929–937 19205856 10.1007/s10803-009-0702-y

[c42] Lord C., Risi S., Lambrecht L., Cook E. H., Leventhal B. L., DiLavore P. C., . . . Rutter M. (2000). The autism diagnostic observation schedule-generic: A standard measure of social and communication deficits associated with the spectrum of autism. Journal of Autism and Developmental Disorders, 30, 205–223 11055457

[c60] Mackinlay R., Kliegel M., & Mäntylä T. (2009). Predictors of time-based prospective memory in children. Journal of Experimental Child Psychology, 102, 251–264 19091327 10.1016/j.jecp.2008.08.006

[c61] Mäntylä T., Carelli M. G., & Forman H. (2007). Time monitoring and executive functioning in children and adults. Journal of Experimental Child Psychology, 96, 1–19 17030038 10.1016/j.jecp.2006.08.003

[c62] Marsh R. L., & Hicks J. L. (1998). Event-based prospective memory and executive control of working memory. Journal of Experimental Psychology: Learning, Memory, and Cognition, 24, 336–349 10.1037//0278-7393.24.2.336 9530843

[c43] Murray D. J. (1967). Role of speech responses in short-term memory. Canadian Journal of Psychology, 21, 263–276 6045500 10.1037/h0082978

[c44] Okuda J., Fujii T., Ohtake H., Tsukiura T., Yamadori A., Frith C. D., & Burgess P. W. (2007). Differential involvement of regions of rostral prefrontal cortex (Brodmann area 10) in time- and event-based prospective memory. International Journal of Psychophysiology, 64, 233–246 17126435 10.1016/j.ijpsycho.2006.09.009

[c45] Otani H., Landau J. D., Libkuman T. M., St. Louis J. P., & Kazen J. K. (1997). Prospective memory and divided attention. Memory, 5, 343–360 9231147 10.1080/741941393

[c46] Raz N. (2000). Ageing of the brain and its impact on cognitive performance: Integration of structural and functional findings In Craik F. I. M. & Salthouse T. A. (Eds.), The handbook of ageing and cognition (2nd ed., pp. 1–90). Hillsdale, NJ: Erlbaum

[c47] Rönnlund M., Mäntylä T., & Nilsson L. (2008). The Prospective and Retrospective Memory Questionnaire (PRMQ): Factorial structure, relations to global subjective memory ratings, and Swedish norms. Scandinavian Journal of Psychology, 49, 11–18 18190398 10.1111/j.1467-9450.2007.00600.x

[c63] Smith G., Della Sala S., Logie R. H., & Maylor E. A. (2000). Prospective and retrospective memory in normal ageing and dementia: A questionnaire study. Memory, 8, 311–321 11045239 10.1080/09658210050117735

[c49] Verfaellie M., Race E., & Keane M. M. (2012). Medial temporal lobe contributions to future thinking: Evidence from neuroimaging and amnesia. Psycholigica Belgica, 52, 77–94 10.5334/pb-52-2-3-77 PMC3581589 23447709

[c50] Volle E., Gonen-Yaacovi G., Costello A., De L., Gilbert S. J., & Burgess P. W. (2011). The role of rostral prefrontal cortex in prospective memory: A voxel-based lesion study. Neuropsychologia, 49, 2185–2198 21371485 10.1016/j.neuropsychologia.2011.02.045 PMC3128701

[c51] Ward H., Shum D., McKinlay L., Baker-Tweney S., & Wallace G. (2005). Development of prospective memory: Tasks based on the prefrontal-lobe model. Child Neuropsychology, 11, 527–549 16306026 10.1080/09297040490920186

[c52] Wechsler D. (1999). Wechsler Abbreviated Scale of Intelligence. New York, NY: Psychological Corporation

[c53] Williams D. (2010). Theory of own mind in autism: Evidence of a specific deficit in self-awareness? Autism, 14, 474–494 20926458 10.1177/1362361310366314

[c64] Williams D., & Happé F. (2009). ‘What did I say?’ versus ‘What did I think?’: Attributing false beliefs to self amongst children with and without autism. Journal of Autism and Developmental Disorders, 39, 865–873 19205861 10.1007/s10803-009-0695-6

[c54] Williams D. M., Boucher J., Lind S., & Jarrold C. (2013). Time-based and event-based prospective memory in autism spectrum disorder: The roles of executive function and theory of mind, and time-estimation. Journal of Autism and Developmental Disorders, 43, 1555–1567 23179340 10.1007/s10803-012-1703-9

[c55] Williams D. M., Bowler D. M., & Jarrold C. (2012). Inner speech is used for short-term memory, but not planning, among intellectually high-functioning adults with autism spectrum disorder. Development & Psychopathology, 24, 224–239 10.1017/S0954579411000794 22293006

[c56] Williams D. L., Goldstein G., Carpenter P., & Minshew N. (2005). Verbal and spatial working memory in autism. Journal of Autism and Developmental Disorders, 35, 747–756 16267641 10.1007/s10803-005-0021-x

[c57] Williams D. M., Payne H., & Marshall C. (2013). Non-word repetition impairment in autism and specific language impairment: Evidence for distinct underlying neuro-cognitive causes. Journal of Autism and Developmental Disorders, 43, 404–417 22733298 10.1007/s10803-012-1579-8

[c65] Wilson B. A. (1991). Long-term prognosis of patients with severe memory disorders. Neuropsychological Rehabilitation, 1, 117–134

[c66] Woodbury-Smith M. R., Robinson J., Wheelwright S., & Baron-Cohen S. (2005). Screening adults for Asperger syndrome using the AQ: A preliminary study of its diagnostic validity in clinical practice. Journal of Autism and Developmental Disorders, 35, 331–335 16119474 10.1007/s10803-005-3300-7

[c58] World Health Organization (1993). International classification of mental and behavioural disorders: Clinical descriptions and diagnostic guidelines (10th ed.) Geneva, Switzerland: World Health Organization

